# Verminous Irritation, a Direct Exciting Cause of Diseases Not Usually Attributed to Any of the Varieties of Intestinal Worms

**Published:** 1845-12

**Authors:** J. C. Scott

**Affiliations:** Rob Roy, Indiana


*Verminous Irritation a direct exciting Cause of Diseases not usually attributed to any of the Varieties of Intestinal Worms.* By Dr. J. C. Scott, of Rob Roy, Indiana.

Case I.—On the 14th April, 1837, I was called to prescribe for a case of erysipelas. This was a well marked case. The inflammation was spread over the arms, shoulders, and face, and the latter was so much tumified as to almost shu the eyes.

The subject of this attack was a little girl, eight years old. There were symptoms of functional disorder of the liver and digestive organs, and I was inclined to refer the attack to this cause. As there was tolerably high arterial action, I had immediate recourse to V. S., and gave an emetic dose of ipecacuana, which operated promptly, bringing off bile.

15th. The emetic given yesterday had produced no discharges from the bowels, and fifteen grs. of calomel, which had been left for the purpose, had been given, and had operated several times. No mitigation of inflammation. Gave small doses of calomel and ipecacuana at appropriate intervals. No external applications were made.

16th. The calomel and ipecacuana had nauseated the stomach, and kept up moderate action of the bowels—discharges green—arterial action a little moderated, but no mitigation of erysipelatous inflammation—face still tumified and eyes almost shut. As the bowels were loose, no purgative medicine was deemed necessary at this time. As an external application, left a weak solution of corrosive sublimate.

17th. Bowels had been moved two or three times during the night, and stomach had been kept a little sick. The solution had been used as directed, but there was as yet no visible change as to inflammation—the face still tumified, and the blotches wearing the same appearance as before. There is, however, this difference. My patient is weaker, and complains of pain in the umbilical region and lower part of the belly. Two dead lumbricoids found in the bed this morning. I had *now* sufficient reason to apprehend the existence of more worms in the bowels, and determined to act immediately in reference to this new indication. Accordingly the spigelia was now given, and followed, after a proper interval, with ol. ricini and sp. terebinth.

18th. The medicine last administered, I conscientiously believe, was the salvation of my patient. It operated well, and I was informed by the mother of the patient, that *forty-five* lumbricoids, of various sizes, were dislodged at *one dejection,* and some of them very large ones. There was now incredible abatement of the erysipelatous inflammation. The tumefaction of the face was rapidly subsiding—the blotches were fast assuming a paler look, and the whole aspect of the case materially changed. Continued the ol. and turpentine in small portions, at proper intervals.

19th. Visited my patient this morning—a few more worms have been discharged. She has some appetite and is fast recovering. This patient was soon restored to good health.

Case II.—On the 26th of June, 1838. I was called to visit Mrs. C., aged 30 years. She has had five healthy children, and at the time of this affliction is nursing the fifth one.

For some time previously she had suffered occasional pain in the ankle of the right leg. She had continued to go about tending to domestic duties, though sometimes with difficulty.

On the day before (25th) the pain became so severe that she could no longer walk or move the joint, and when I arrived on the morning of the 26th., the ankle was much swelled, inflamed, and tender. She would scarcely be prevailed upon to suffer it to be examined. The pulse was hard and quick; tongue moderately coated; complained of shooting pain through the head, and there was intolerance of light. Her husband informed me that the day before, she had been seized with a "jerking," and, as she herself expressed it, "very curious feelings all over," which lasted several hours. This intelligence gave me the idea that the convulsive muscular action might depend on local irritation, and (I should have mentioned before that this patient had been repeatedly treated for chronic affection of the liver) an irregular condition of the bowels. (Dr. Armstrong, I think, ascribes chorea to the latter condition of the small intestines, and also of the colon and liver.) I bled the patient freely, and after unloading the bowels, gave alterative portions of calomel suitably combined—made the usual applications to the affected joint, and throughout prescribed as for a well defined case of acute rheumatism. All the medicine seemed to act very well, with but this exception; healthy bile, so far, was out of the question. A blister applied to the lame ankle, had the effect to reduce the swelling in about the proportion of the amount of serum discharged from the blistered part, and did, certainly, in no inconsiderable degree, relieve the pain, but then, there appeared to be paralysis of the limb to a remarkable extent. At first I was of opinion that instead of inability to move the limb, there was want of inclination to do so, from soreness of the part occasioned by the blistering, as also from recollection of pain previously experienced on the slightest motion; but, upon closer examination, I found I was mistaken.

Such is the history of this case up to the 30th. About this time several lumbricoid worms were seen in the dejections.

I was yet an inexperienced practitioner, and did not at all suspect what I now believe to have been the true source of this pain, &c. And besides, not having as yet met with disease in an adult person, the cause of which might be referred to verminous irritation, I was hardly inclined to think that worms had anything to do with the case. Therefore I had no resourse to anthelminties, (unless, indeed, calomel may be so denominated) further than to give a single dose of ol. ricini and sp. terebinth, with the design to work off some calomel, which had been taken in combination with pulv. dov. I often use ol. and turpentine as a physic. I think the combination an excellent one for this purpose. Still, however, the worms continued to come off with the stools, until some fifteen or twenty had escaped; and from this time my patient began to improve. She could now move the leg without the *least pain*, and was perfectly well in a few days.

I am now decidedly of opinion that this was a case of rheumatism, having for its direct, exclusive, exciting cause, *verminous irritation.*

In reference to these two cases, as also to others of striking similitude, one question naturally arises—are they strictly referable to verminous irritation, as the exclusive, *exciting* cause? or, on the contrary, does the irritation produced by these "parasitic animals," as they are sometimes called, only simulate such and such diseases? I think the question is now fairly stated.

When we consider the number and complexity of the sympathies existing between the alimentary canal and the different parts of the body, we are at a loss to determine, in very many instances, what particular modes of irritation influence the various phenomena of diseases. Who then can say, with positive assurance, that the kind of irritation we are now considering, is not sometimes the exclusive, exciting cause of certain diseases not attributed in the books to such irritation? In the cases above mentioned, (and since these occurred I have met with some two or three similar ones) the disease was not broken up, until the expulsion from the bowels of a number of worms; and when this took place the relief was prompt and perfect. The cause of irritation in the intestinal canal was removed, and when this was the case, disease yielded. I therefore, think, to say the least that ought to be said in relation thereto, that this kind of an irritation was an exciting cause of the disease, if not the *exclusive* cause. However much the fastidious critic may be inclined to sneer at she notion, I, for one, cannot consent to the contrary hypothesis of mere *simulation*, until he shall furnish me an infallible symptomatology of worms.

I have no where seen a case of erysipelas reported, the cause of which was referred to verminous irritation; but considering the very intimate sympathy between the intestinal canal and that extremely sensitive and important integument of the human body, the skin, it would not be wonderful should such a disease have occurred, and that too, from irritation. And if from irritation, why not from verminous irritation? But an author of high reputation, both as a writer and teacher of medicine, (the late Dr. Eberle) has left his testimony on this subject to the following effect. "Chorea, epilepsy, hydrocephalus, emaciation, convulsions, paralysis, fevers, dropsy and a vast variety of anomalous afflictions, are at times the immediate consequences of verminous irritation, and frequently disappear after the expulsion of the worms." The same author says of Esquirol that he "knew eleven persons *cured* of mania by the expulsion of a large number of lumbrici." And Brera is represented to state that "pains in the joints, similar to those of arthritic rheumatism, were occasioned by worms in the intestinal canal. The pains immediately ceased upon the expulsion of *nine* large lumbricoides."

After all this, and much more that might be said on this interesting subject, I do not see the necessity of the doctrine of "simulation," as it is called by some.

The preceding narration of cases, and the few brief and imperfect remarks I have made in relation thereto, are prompted more particularly by an article on this subject, which made its appearance a few years ago in the Transylvania Journal of Medicine, vol. 10, p. 788.

The communication of Dr. Scott proceeds to quote from a paper by W. Markley Lee, M. D., published as cited, and to comment thereon. The amount of other matter in type before the arrival of Dr. S.'s paper, compels us to omit that portion of his essay, and refer our readers to the paper itself, with which they may compare the position assumed and discussed by Dr. Scott. Ed.

